# Microleakage after Thermocycling of Three Self-Etch Adhesives under Resin-Modified Glass-Ionomer Cement Restorations

**DOI:** 10.1155/2010/728453

**Published:** 2010-06-06

**Authors:** Sabine O. Geerts, Laurence Seidel, Adelin I. Albert, Audrey M. Gueders

**Affiliations:** ^1^Division of Conservative and Adhesive Dentistry, Department of Dentistry, University of Liège, Box 45, 4020 Liège, Belgium; ^2^Department of Biostatistics, University of Liège, Box 45, 4020 Liège, Belgium

## Abstract

This study was designed to evaluate microleakage that appeared on Resin-Modified Glass-Ionomer Cement (RMGIC) restorations. Sixty class V cavities (h × w × l = 2 mm × 2 mm × 3 mm) were cut on thirty extracted third molars, which were randomly allocated to three experimental groups. All the buccal cavities were pretreated with polyacrylic acid, whereas the lingual cavities were treated with three one-step Self-Etch adhesives, respectively, Xeno III (Dentsply Detrey GmbH, Konstanz, Germany), iBond exp (Heraeus Kulzer gmbH & Co. KG, Hanau, Germany), and Adper Prompt-L-Pop (3M ESPE AG, Dental products Seefeld, Germany). All cavities were completely filled with RMGIC, teeth were thermocycled for 800 cycles, and leakage was evaluated. Results were expressed as means ± standard deviations (SDs). Microleakage scores were analysed by means of generalized linear mixed models (GLMMs) assuming an ordinal logistic link function. All results were considered to be significant at the 5% critical level (*P* < .05). The results showed that bonding RMGIC to dentin with a Self-Etch adhesive rather than using polyacrylic acid did not influence microleakage scores (*P* = .091), except for one tested Self-Etch adhesive, namely, Xeno III (*P* < .0001). Nevertheless, our results did not show any significant difference between the three tested Self-Etch adhesive systems. In conclusion, the pretreatment of dentin with Self-Etch adhesive system, before RMGIC filling, seems to be an alternative to the conventional Dentin Conditioner for the clinicians as suggested by our results (thermocycling) and others (microtensile tests).

## 1. Introduction

Improvements in the techniques of adhesive dentistry have allowed the use of minimal invasive cavities and aesthetic fillings. During the past few years, adhesive bonding systems have submitted major developments and the durability of adhesive restorations has continually grown [1]. Resin-composites and Glass Ionomer Cements (GICs) have also been considerably improved in their aesthetic and mechanical properties. Moreover, in the long term, composites have demonstrated better mechanical performances and surface integrity than GICs or Resin-Modified Glass Ionomer Cements (RMGICs) [2–5]. In addition, composites are more aesthetic and polish better than GICs or RMGICs. Nevertheless, RMGICs show many advantages [6, 7]. Firstly, RMGICs allow an optimal sealing on the marginal join by a quasitotal lack of microleakage [4, 8, 9]. Secondly, this material (RMGIC) is more tolerant to moisture than resin composites, and thus, it does not require a rubber dam [10–13]. Thirdly, its use is reported with very few cases of postoperative sensitivity [4, 14, 15]. Fourthly, GICs and RMGICs are able to release fluorides, which induce remineralization of the surrounding calcified dental tissues [16–19]. Fifthly, several studies have shown that RMGICs self-adhere to dental tissues but this level of adhesion was shown to be less than that obtained by composite restorations bonded with adhesive systems [20–23]. Taking this into account, some authors have tested RMGICs bonded to dentin with a Self-Etch adhesive system (SE) and their results have shown an enhancement of bond strength [21, 22, 24]. Nevertheless, when the RMGIC was bonded to dentin with an adhesive system, the self-adhesion properties of the ionomer cement were not expressed: for instance, it was supposed that a surrounding dental tissues remineralization and an optimal sealing of dentinal tubuli would not appear. But, a good hermeticity of the filling seems to be an important factor for clinicians. So, the aim of our study was to evaluate the microleakage (rather than mechanical bond strength) that was permitted by different RMGIC restorations: RMGIC was placed on the dentin after application of either polyacrylic acid or self-etch adhesive bonding systems. 

In this study, we examined the null hypothesis that bonding RMGIC to dentin with a self-etch adhesive did not improve the marginal sealing.

## 2. Materials and Methods

All tested teeth in our study were extracted for medical reasons. This explains the unnecessary approval by ethical committee. However, all subjects were orally informed that the extracted teeth could be included in an experimental study. 

Thirty recently extracted third molars without decay were stored in refrigerated saline solution for maximum 3 months as recommended by the ISO norms (ISO. Guidance on testing of adhesion to tooth structure. International Organization for Standardization. TR 11405,1–4, Geneva, Switzerland, 1994). On each tooth, two rectangular cavities (h × w × l = 2 mm × 2 mm × 3 mm) were prepared at the cemento-enamel junction with a cylindrical diamond drill (diameter = 0,9 mm). The margins of the cavities were butt-jointed, half in the enamel and half in the root dentin. Teeth were randomly and equally allocated to three groups of tested restorations (for each group, the number of teeth was 10 and the number of cavities was 20). 

The procedures for the pretreatment of dental tissues and for the filling of cavities are summarized in Table 1. 

The composition of main materials that were used in this study was displayed in Table 2.

The buccal cavities were pretreated 10 seconds with 10% polyacrylic acid (DC) (Dentin Conditioner, GC, Tokyo, Japan) and were filled with an RMGIC (FII) (Fuji II LC, GC, Tokyo, Japan). The lingual cavities were pretreated with three different Self-Etch (SE) adhesive systems before filling with the same RMGIC as on the buccal side.

- In the first group (group I), cavities were pretreated with Xeno III (XIII) (Dentsply Detrey GmbH, Konstanz, Germany), an Intermediary Strong Self-Etch (ISSE, pH of approx. 1.5).
- In the second group (group II), cavities were pretreated with experimental iBond (iB exp) (Heraeus Kulzer GmbH & Co. KG, Hanau, Germany), an Intermediary Strong Self-Etch (ISSE, pH of approx. 1.5).
- In the third group (group III), cavities were pretreated with Adper Prompt-L-Pop (APLP) (3M ESPE AG, Dental products, Seefeld, Germany), a Strong Self-Etch (SSE), which presented a pH of below 1.0.

Polyacrylic acid and adhesives were used according to the manufacturer's instructions. Photopolymerization was carried out with the same halogen lamp (XL 3000, 3M ESPE AG, Dental products, Seefeld, Germany):

- XIII was cured during 10 seconds,
- iB exp was cured during 20 seconds,
- APLP was cured during 10 seconds,
- Fuji II was cured during 20 seconds.

The output of the light was checked with a radiometer and we assume that our halogen lamp had at least 400 mW/cm^2^ during all the experimentations. 

The preparations were finished with diamond drills and polished with disks (Hawe Neos Dental, Bioggio, Switzerland) under water-spray.

**Figure 1:**
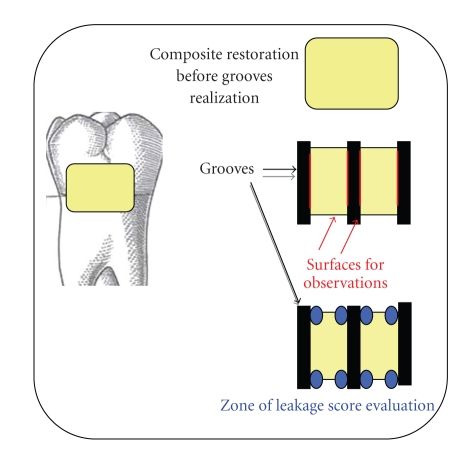
Illustration of the 3 grooves cut on the composite filling and the resulting 8 lecture areas.

After that, the apexes were fixed in an autopolymerizing resin (Paladur, Heraeus-Kulzer GmbH & Co. KG, Hanau, Germany) and the specimens were immersed in saline solution for twelve weeks (in a refrigerator at 5°C). Thereafter, they were thermocycled (5°C–55°C) for 800 cycles in 22 hours. After thermocycling, the teeth were immersed in silver nitrate solution (6 hours) and in 25% vitamin C during 10 minutes (pH about 3) [25, 26]. After immersion, the samples were prepared with three grooves in the restoration. The interfaces occurred between the teeth and the filling have been described in our previous study [27]. Briefly, a cylindric diamond drill (0,9 mm diameter) was placed perpendicular to the restoration and 3 grooves (3 mm depth) were cut: one at the mesial margin, one at the distal margin, and one right in the middle of the filling (Figure 1). These preparations yielded four evaluating surfaces for each preparation, for a total of 240 viewing surfaces. Each sample allowed one measure in enamel and one in dentin (lecture areas), for a total of 480 measures, 160 for each group.

Each section was examined by twofold magnification by means of an optic microscope (Carl Zeiss, SAS, Oberkochen, Germany) and the observation of each tooth was made twice. All samples were observed by the same operator (blinded test).

**Figure 2:**
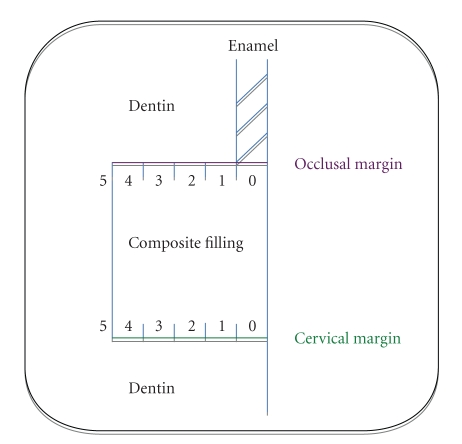
Illustration of the 6-point severity scale used to evaluate the microleakage at the margins of the restorations (occlusal and cervical margins).

Arbitrarily, the evaluation of leakage was made with a 6-point severity scale (Figure 2) [27].

 Score = 0: no leakage.
 Score = 1: leakage up to the enamel-dentin junction or a depth of 0.5 mm on the radicular wall.
 Score = 2: leakage up to the maximum half of the lateral wall (leakage depth ≤1 mm).
 Score = 3: leakage over half of the lateral wall (1 mm < leakage depth < 2 mm).
 Score = 4: subtotal leakage on the whole of the lateral wall (leakage depth = 2 mm).
 Score = 5: total leakage partly or entirely on the pulpal wall of the cavity (leakage depth > 2 mm).

We postulated that higher scores of microleakage (scores 3, 4, and 5) after thermocycling would be responsible for clinical failure of the bonding.

### 2.1. Statistical Analysis

Results are expressed as means ± standard deviations (SDs). Microleakage scores were analysed by means of generalized linear mixed models (GLMMs) assuming an ordinal logistic link function. Covariates in the model were (1) pretreatment of dental tissues (polyacrylic acid/Self-Etch adhesive system) and (2) interface (enamel or dentin). The model also accounts for repeated measurements on the various teeth. All results were considered to be significant at the 5% critical level (*P* < .05). Statistical calculations were made using the SAS (version 8.2 for Windows) package.

## 3. Results

As seen in Table 2, there is no significant difference between either technique of RMGIC restoration (*P* = .091): respectively, for all buccal (RMGIC restorations using with a polyacrylic acid) and lingual (RMGIC restorations using with an SE adhesive) filled cavities, the mean scores of microleakage were 0.91 ± 0.88 and 0.77 ± 0.78. Furthermore, there is no significant difference between the buccal and the lingual restorations from group II (DC-2 + FII-2 versus iB exp + FII, *P* = .43), or from group III (DC-3 + FII-3 versus APLP + FII) (*P* = .33). In group I, the microleakage of RMGIC used with Dentin conditioner was the highest score that we have observed in the present study. Nevertheless, in group I, the mean scores of microleakage were significantly different on the buccal (1.49 ± 0.94) from on the lingual sides (0.81 ± 0.76) (*P* < .0001). Also, results showed that there is no significant difference between the 3 different one-step SE adhesives used to bond the RMGIC.

## 4. Discussion

It can be assumed that silver nitrate penetration is a harsh test of the marginal seal because the size of silver ions (nanoleakage scale) is smaller than that of bacteria (microleakage scale). This suggests that less leakage may have occurred in vivo than in vitro [28]. Taking this into account, data displayed in Table 3 show small leakage scores in all tested cases suggesting that all tested adhesive materials are efficient. Unfortunately, the standard deviations were important and suggested the technique sensitivity of the adhesive systems as described by others [29–31].

As discussed in our previous study [27], thermocycling is the only in vitro test for simulating thermal stress in teeth [28, 32, 33]. Ideally, filling materials and dental tissues should have identical coefficients of thermal expansion in order to limit leakage at the margins of the restorations [34–38]. In fact, these coefficients are similar for both dentin and RMGIC [39], while they differ significantly between dentin and composites [9]. So, the well-described microleakage of composite restorations results from polymerization shrinkage and/or from the difference between thermal expansion coefficients. Microleakage complications include many postoperative failures such as sensitivity, recurrent caries, pulp inflammation, and necrosis [40, 41]. In this way, RMGICs have gained favour because of their excellent ability to decrease postoperative sensitivity and their capacity to release fluoride [19]. In addition, RMGICs have been recognized to create less stress on the residual cavity walls and to improve marginal adaptation [20] because of the material's favourable visco-elastic properties [8]. In fact, RMGIC is a good substitute for dentin, particularly in deep cavities; however, it does not provide a good replacement for enamel because of its poor durability [3, 9, 20, 42]. For this reason, RMGIC is often used in the sandwich technique, where enamel is replaced by composite material [3, 9], or in class V cavities. 

Data from the literature have shown that the presence of a smear layer can interfere with the adhesion of RMGIC to dentin [43] and that this smear layer can break cohesively during shrinkage polymerization [44]. So, several authors have reported an improvement in the bond strength of RMGIC to dental tissues after pretreatment with polyacrylic acid, which is able to remove the smear layer [45–47] and can partially demineralize the dentin surface [45, 48, 49]. Since the major characteristic of RMGIC is that this material contains HEMA, it was recently found that bonding RMGIC with a composite adhesive system, namely, a self-etch adhesive, enhanced significantly the dentin shear bond strength of this hybrid material (tensile test) [21, 22, 50]. Thus, we have supposed that this adhesive resin bonding procedure could permit less microleakage than a dentin treatment with conventional polyacrylic acid. Results from our microleakage experiment do not support this hypothesis, since we showed that bonding RMGIC with an SE adhesive rather than using a dentin conditioner did not improve the mean scores of microleakage, except in the case of sample XIII (group I). This significant difference between test (Dentin conditioner) and control groups (self-etch adhesives) was only observed for one of the three groups, namely, group I. We can assume that this was due to a failure in operator handling because similar procedures were conducted in the other two control groups (group II: DC-2 + FII-2; group III: DC-3 + FII-3): both of these control groups presented lower mean scores of microleakage in comparison with the group I. In fact, in our specimens in the group I, only 2 of the 10 tested buccal restorations showed at least very important microleakage (scores of 3 and 4), while the 8 others did not. 

## 5. Conclusion

This study was designed to test the null hypothesis that bonding RMGIC to dentin with an one-step adhesive system would not enhance the performance of this hybrid material. Our results support this null hypothesis, but the pretreatment of dentin with an SE adhesive before RMGIC filling seems to be an alternative to the conventional Dentin Conditioner, as suggested by other authors [50]. Nevertheless, the results of our in vitro study need some further clinical investigations.

## Figures and Tables

**Table 1:** Pretreatment and bonding procedures for the 3 groups tested.

Group	Buccal Cavity (*n*)	Lingual Cavity (*n*)
(Number of cavities)		
Group I	DC-1 + FII-1 (10)	XIII + FII (10)
(*n* = 20)		
Group II	DC-2 + FII-2 (10)	iB exp + FII (10)
(*n* = 20)		
Group III	DC-3 + FII-3 (10)	APLP + FII (10)
(*n* = 20)		

DC: Dentin Conditioner (GC Tokyo, Japan).

FII: Fuji II LC (GC Tokyo, Japan).

XIII: Xeno III (Densply Detrey GmbH, Konstanz, Germany).

iB exp: iBond experimental (Heraeus Kulzer GmbH & Co. KG, Hanau, Germany).

APLP = Adper Prompt-L-Pop (3M ESPE AG, Dental products, Seefeld, Germany).

**Table 2:** Composition of biomaterials used in the present study.

Biomaterials	Components
Dentin conditioner	– Distilled water (90%)
(GC Tokyo, Japan)	– Polyacrylic acid (10%)

	Liquid:
	– Distilled water
	– Polyacrylic acid
Fuji II LC	– 2-hydroxyethyl methacrylate (HEMA)
(GC Tokyo, Japan)	– Urethane dimethacrylate
	– Camphorquinone
	Powder:
	Fluoro alumino silicate glass

	Liquid A:
	– 2-hydroxyethyl methacrylate (HEMA)
	– Purified water
	– Ethanol
	– Butylated hydoxy toluene (BHT)
	– Highly dispersated silicon dioxide
Xeno III	Liquid B:
(Densply Detrey GmbH, Konstanz, Germany)	– Phosphoric acid modified methacrylate (Pyro-EMA)
	– Mono fluoro phosphazene modified methacrylate
	– Urethane dimethacrylate
	– Butylated hydoxy toluene (BHT)
	– Camphorquinone
	– Ethyl-4-dimethylaminobenzoate

iBond experimental	Unknown
(Heraeus Kulzer GmbH & Co. KG, Hanau, Germany)	

	Liquid 1 (red blister):
	– Methacrylated phosphoric esters
	– Bis-GMA
	– Camphorquinone
Adper Prompt-L-Pop	– Stabilizers
(3M ESPE AG, dental products, Seefeld, Germany)	Liquid 2 (yellow blister):
	– Water
	– 2-hydroxyethyl methacrylate (HEMA)
	– Polyalkenoic acid
	– Stabilizers

**Table 3:** Mean scores of microleakage for RMGIC restorations using a polyacrylic acid conditioning or three different self-etching adhesive systems.

	Mean scores of microleakage (±SD)				*P*
	DC + FII		SE + FII		
GROUP I	DC-1 + FII-1	1.49 (±0.94)	XIII + FII	0.81 (±0.76)*	<.0001
	(*n* = 10)		(*n* = 10)		
GROUP II	DC-2 + FII-2	0.64 (±0.68)	iB exp + FII	0.81 (±0.93)*	.43
	(*n* = 10)		(*n* = 10)		
GROUP III	DC-3 + FII-3	0.61 (±0.70)	APLP + FII	0.69 (±0.63)*	.33
	(*n* = 10)		(*n* = 10)		

Total	All DC + FII	0.91 (±0.88)	All SE + FII	0.77 (±0.78)	.091
	(*n* = 30)		(*n* = 30)		

*When the different adhesive systems are compared, there is no statistically significant difference between the 3 one-step SE adhesives tested in this study (*P* = .73).
